# Development of Chitosan/Gelatin-Based Hydrogels Incorporated with Albumin Particles

**DOI:** 10.3390/ijms232214136

**Published:** 2022-11-16

**Authors:** Magdalena Bańkosz

**Affiliations:** Department of Materials Engineering, Faculty of Materials Engineering and Physics, Cracow University of Technology, 37 Jana Pawła II Av., 31-864 Krakow, Poland; magdalena.glab@doktorant.pk.edu.pl

**Keywords:** hydrogel materials, albumin carriers, swelling properties, release of protein, wound healing

## Abstract

The research subject of this paper are natural polymer-based hydrogels modified with albumin particles. The proteins were obtained via the salt-induced precipitation method, and next characterized using dynamic light scattering (DLS), UV-Vis spectroscopy and FT-IR spectroscopy. The most favorable composition showing monodispersity and particles with a size lower than 40 nm was selected for modification of hydrogels. Such systems were obtained via the photopolymerization performed under the influence of UV radiation using diacrylate poly(ethylene glycol) as a crosslinking agent and 2-hydroxy-2-methylpropiophenone as a photoinitiator. Next, the hydrogels’ swelling ability, mechanical properties, wettability and surface morphology were characterized. Moreover, FT-IR spectroscopy, incubation studies in simulated physiological liquids, pro-inflammatory activity analysis and MTT reduction assay with L929 murine fibroblasts were performed. The release profiles of proteins from hydrogels were also verified. Materials modified with proteins showed higher swelling ability, increased flexibility even by 50% and increased surface hydrophilicity. Hydrogels’ contact angles were within the range 62–69° while the tensile strength of albumin-containing hydrogels was approx. 0.11 MPa. Furthermore, the possibility of the effective release of protein particles from hydrogels in acidic environment (approximately 70%) was determined. Incubation studies showed hydrogels’ stability and lack of their degradation in tested media. The viability of fibroblasts was 89.54% for unmodified hydrogel, and approx. 92.73% for albumin-modified hydrogel, and such an increase indicated the positive impact of the albumin on murine fibroblast proliferation.

## 1. Introduction

Albumin is a non-toxic, natural polymer showing excellent biocompatibility [1]. This protein is also characterized by stability, biodegradability, high solubility and, importantly, ligand-binding properties [2,3]. These characteristics result in growing interest in the use of this substance for development of various materials designed for biomedical purposes [4]. Albumin finds application for example in tissue engineering [5,6,7] or as a coating of implant surfaces [8,9].

The highest potential of albumin is meanwhile noticeable in designing drug delivery systems. Its abundant accumulation both in benign and malignant lesions, long half-life and non-immunogenicity [10,11] as well as the presence of multiple sites able to bind with various molecules [12,13,14] make it a promising material as carrier for targeted delivery of active substances. It was also demonstrated that albumin protects drugs both from metabolism and elimination in vivo which also enables their delivery to the specific place [15]. For example, Rejinold et al. performed investigations on bovine serum albumin (BSA) coated nanoparticles as carriers of niclosamide, i.e., a drug showing potential for COVID-19 treatment [16]. In turn, Deng et al. developed albumin-based hydrogels incorporated with dihydromyricetin, i.e., a natural active substance showing anti-inflammatory, antibacterial and neuroprotective properties. It was proved that the hydrogels were biocompatible, were desirable mechanical properties and, importantly, showed an ability to sustained drug release [17]. The importance of albumin-based carriers is being particularly noticed in the case of delivery of anticancer drugs [18]. It was reported that albumin acts as a nutrient source by cancer cells, and this is the reason why this protein shows accumulation to a greater extent near these cells than the healthy ones [19]. Therefore, many studies are currently being performed to develop albumin-based carriers of chemotherapeutics and other albumin-based biomaterials for cancer treatment [20,21,22,23].

The most common method of the preparation of albumin carriers is the desolvation method which requires the use of a dehydrating agent such as ethanol which removes the hydrated membrane of albumin and thereby allows for its precipitation. However, in order to obtain stable protein particles, it is necessary to additionally use a crosslinking agent, e.g., glutaraldehyde. The desolvation method is the most commonly used method of obtaining protein particles due to its relative simplicity and quick course. However, a significant disadvantage of this method is the problem with the removal of glutaraldehyde and organic dehydrating agent residues which may cause a toxic effect on the organism [24]. Among other methods of obtaining albumin particles such may be distinguished as e.g., emulsification, i.e., the method consisting in the use of an oil phase and an albumin solution acting as a water phase, their mixing and subsequent intensive stirring enabling the formation of an emulsion. The disadvantage of this method is the necessity to stabilize the emulsion via the thermal or chemical treatment [25]. Other methods are e.g., the thermal gelling or nanospray (due to the necessity of the use of high temperature condition they may lead to the denaturation, and thus the inactivation of albumin) as well as nanoparticle albumin bound (NAB) technology which in turn requires the use of toxic organic solvents such as chloroform or dichloromethane [26]. Due to the mentioned disadvantages of commonly used methods of obtaining albumin particles, in this work a newly developed method was applied which was described in detail in the previous work [27]. The developed method is innovative—it is simple and quick and, importantly, there is no need to use organic compounds showing toxic activity. The purpose of the work was to combine the albumin nanoparticles obtained as a result of the developed method with a hydrogel material that could be applied as innovative III-generation dressing material.

There are many works presenting chitosan-based biomaterials incorporated with albumin. For example, Li et al. developed chitosan and alginate-based nanocomplexes acting as BSA carriers [28]. Next, Kim et al. investigated hydrogels based on the combination of glycerol phosphate disodium salt and chitosan and obtained via the electrostatic crosslinking. The studies were conducted to verify the potential of these systems as vehicles for BSA—fluorescein isothiocyanate [29]. In other work, UV-photopolymerizable chitosan-based hydrogels incorporated with albumin carriers and also with *Aloe vera* juice were characterized in detail [30]. Nonetheless, to my knowledge there were no studies focused on albumin particles containing hydrogels based on gelatin and chitosan. However, the literature reports indicate a high potential of chitosan/gelatin hydrogels as dressing materials [31]. For example, Magli et al. developed chitosan/gelatin-based 3D-printed hydrogels via the chemical modification using methylfurfural [32]. In turn, Ferreira et al. characterized chitosan/gelatin hydrogels modified with phlorotannin obtained by means of the electrospinning. Such obtained materials showed no cytotoxic activity towards hDNF cells thus enabling their adequate adhesion and proliferation [33]. Next, it was demonstrated that chitosan/gelatin hydrogel dressings may affect adversely the microbes, support cell proliferation and neovascularization as well as induce granulation tissue formation supporting the same wound healing processes [34]. Due to the high application potential of chitosan/gelatin hydrogels these polymers were selected as base components of developed materials. Thus, the main purpose of the work was to develop hydrogel dressing based on such natural polymers as chitosan and gelatin and modified additionally with albumin particles prepared via the salt-induced protein precipitation process. The presented combination has not been described in the literature so far. Moreover, the method of the protein particle synthesis is also a newly developed technique.

Thus, in this paper, albumin carriers were obtained via the salt-induced precipitation technique and characterized using dynamic light scattering (DLS), UV-Vis spectrophotometry and Fourier transform infrared (FT-IR) spectroscopy. Next, chitosan/gelatin-based hydrogels containing protein-based carriers were obtained using UV radiation. Further research aimed at analyzing the physicochemical properties of prepared polymers including determining their swelling capability, characterizing their chemical structure via FT-IR technique and verifying their tendency to degradation in simulated physiological environments. Furthermore, hydrogels’ mechanical properties were investigated, and their surface morphology was evaluated via scanning electron microscopy (SEM). Finally, the hydrogels’ wettability and their ability to sustained albumin releasing were also determined.

The main application purpose of the developed materials is their potential use as dressing materials supporting cancer treatment from the outside, i.e., as materials applied to the skin in the case of skin cancers. Albumin, as it was previously mentioned, is an excellent carrier of chemotherapeutic drugs while the hydrogel dressing supports the regenerative processes of wounds formed around the neoplastic tissue. Due to the target biomedical application, performed studies included also in vitro biological investigations using L929 murine fibroblasts as well as determining the pro-inflammatory activity of developed hydrogels.

## 2. Results and Discussion

### 2.1. Characterization of Protein Particles

#### 2.1.1. Optical Properties of Protein Particles Verified via the UV-Vis Spectroscopy

**Figure 1:**
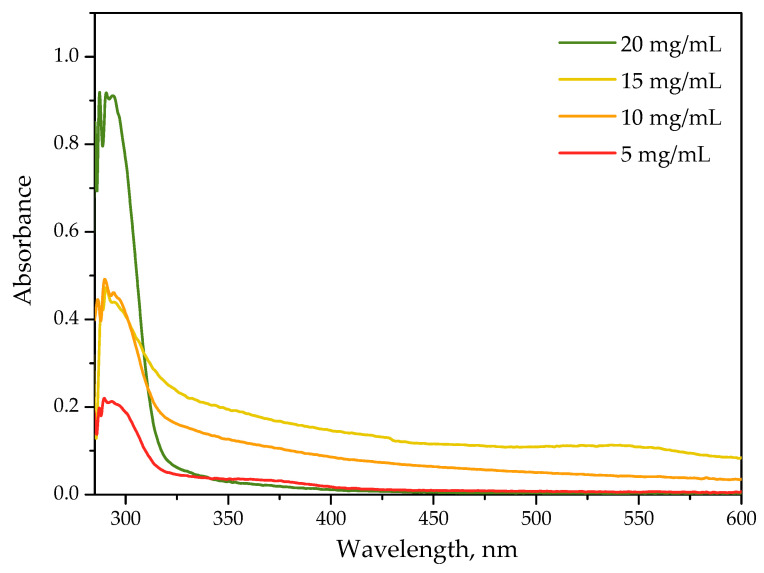
UV-Vis spectra of protein particles suspensions obtained using various concentration of albumin solution.

Results of UV-Vis spectroscopy of protein particles obtained using albumin solution at varying concentrations are shown below in Figure 1.

Albumin is a protein consisting of a row of amino acids including aromatic ones. Thus, the aromatic rings included in the structure of this protein are responsible for its optical properties. According to the literature reports, the adsorption bands characteristic for π → π* transitions of the phenyl rings of tryptophan, tyrosine and phenylalanine occur within the range 270–290 nm [35]. On presented above UV-Vis spectra of all tested compositions, the adsorption band with a maximum at approximately 280 nm characteristic for albumin may be observed. The individual bands differ in their intensities. The highest intensity was observed for protein particles obtained using albumin solution with the highest concentration, i.e., 20 mg/mL wherein the lowest one was noticed for the particles prepared using albumin solution with the lowest concentration, i.e., 5 mg/mL.

#### 2.1.2. Results of Spectroscopic Analysis of the Protein Particles

**Figure 2:**
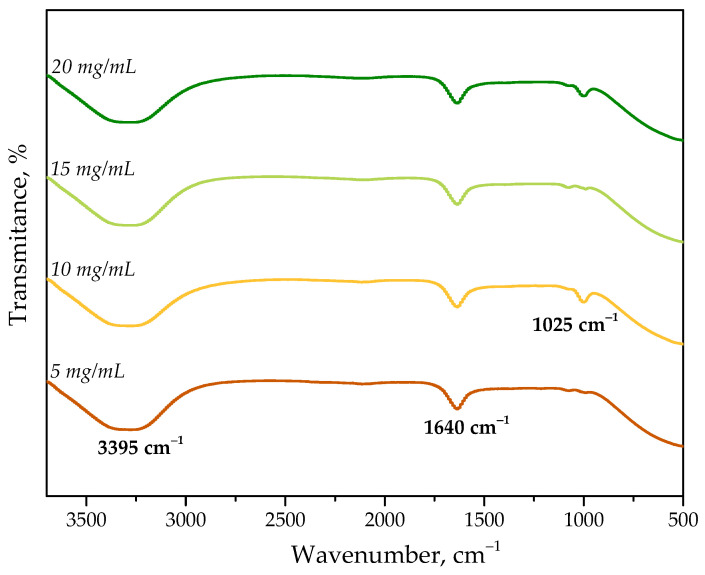
FT-IR spectra of protein particles suspensions obtained using various concentration of albumin solution.

FT-IR spectra of all obtained compositions are presented below in Figure 2.

FT-IR spectroscopy confirmed the occurrence of the absorption bands characteristic for applied protein. The wide absorption band with a maximum at approximately 3395 cm^−1^ may be assigned to the stretching vibrations of -OH and -NH_2_ groups. Next, the band visible at approximately 1640 cm^−1^ corresponds to the vibrations of amide I bond while the band observed at 1025 cm^−1^ is characteristic for the stretching vibrations of C-O bond [36,37,38]. All mentioned absorption bands are characteristic for albumin and thus confirm its presence as it was in the case of the results of UV-Vis spectroscopy.

#### 2.1.3. Results of DLS Analysis

**Figure 3:**
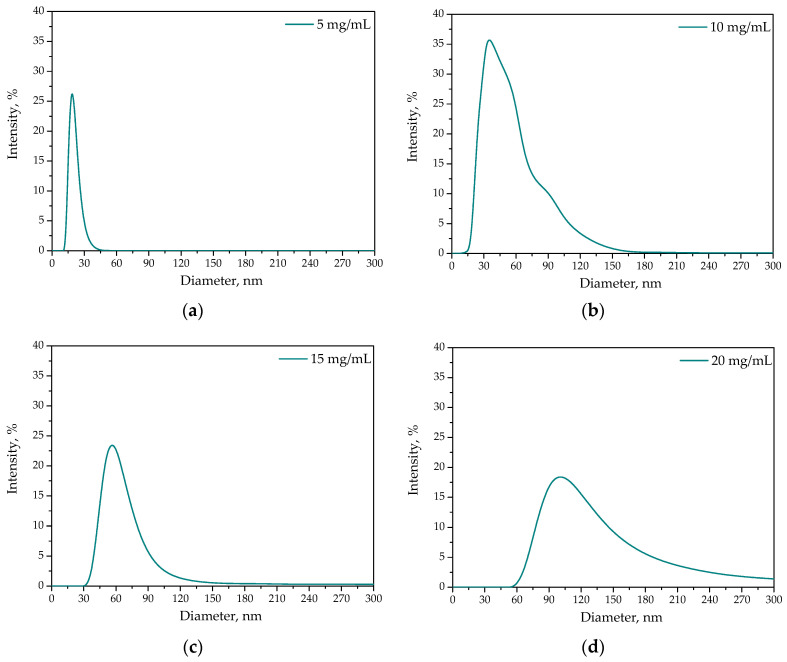
Results of DLS analysis of protein particles obtained using albumin solution at a concentration of 5 mg/mL (**a**), 10 mg/mL (**b**), 15 mg/mL (**c**) and 20 mg/mL (**d**).

Results of DLS analysis are presented in Figure 3. Performed measurements allowed to determine the average hydrodynamic diameter of protein particles obtained using albumin solutions of varying concentrations.

Based on the results of DLS analysis it may be noticed that as the concentration of albumin solution used during the synthesis of protein particles increased, these particles’ sizes also increased. Moreover, in the case of the particles obtained using albumin solutions with concentrations of 10 mg/mL and above, their polydispersity also increased. Probably the use of too-high protein concentration led to the simultaneous precipitation of many albumin particles which, in excess, may agglomerate into larger clusters. As a result, the increase in the particle size with an increase in the protein concentration used as well as a simultaneous increase in the polydispersity of the obtained systems may be observed. The lowest polydispersity—so narrow size distribution—was reported for the particles obtained using albumin solution at a concentration of 5 mg/mL. Such obtained protein suspensions were monodispersed and contained only nanosized particles (with sizes below 40 nm). Thus, due to the narrow size distribution and nanometric sizes, the particles obtained using 5 mg/mL albumin solution.

### 2.2. Characterization of Chitosan/Gelatin Based Hydrogels Incorporated with Protein Particles

#### 2.2.1. Analysis of Hydrogels’ Swelling Capability

**Figure 4:**
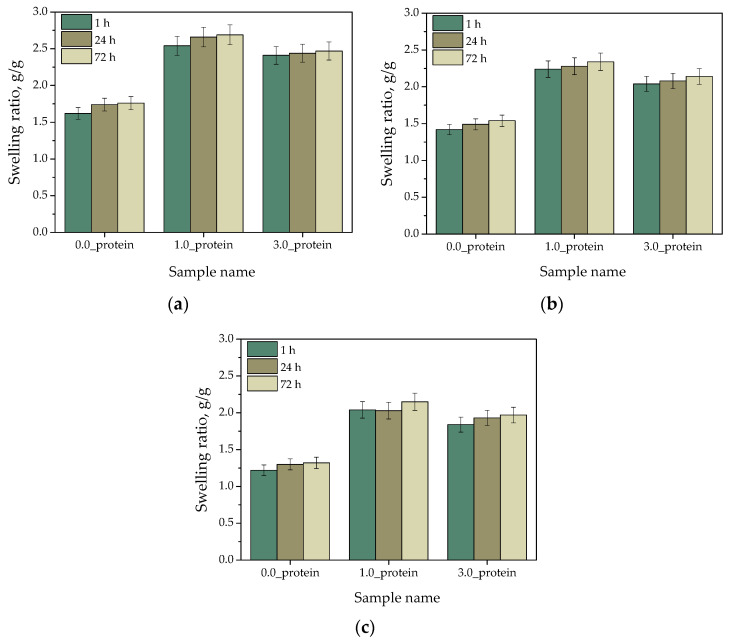
Results of swelling studies in distilled water (**a**), Ringer liquid (**b**) and SBF (**c**) (*n*—number of repetitions, *n* = 3).

In Figure 4, the results of hydrogels’ swelling in selected simulated body liquids are presented. The studies were carried out in triplicates, and their results are shown as average values from performed measurements with corresponding standard deviations (SD, given as error bars).

One of the most important properties of hydrogels is their swelling ability. These materials are able to absorb and retain in their structure large quantities of water without simultaneous degradation or dissolution of polymer network. When a hydrogel is in contact with absorbed liquid, the liquid molecules interact firstly with an outer hydrogel surface. Then, the polymer chains within the polymer network are successively loosened, and the liquid molecules penetrate deeper and deeper into the polymer matrix thus penetrating the interior of the hydrogel network. This phenomenon is very often associated with simultaneous release of modifying substances (such as e.g., drugs) placed between polymer chains. The material swelling with time as a result of the loosening of the polymer network is capable of releasing the residual substances which are pushed out by the penetrating liquid molecules. Thus, determining the swelling properties of hydrogels is a very important aspect in terms of their physicochemical characterization. The swelling capacity versus time for a given hydrogel sample is obtained by the performing the swelling measurements—thus the calculating the swelling ratio Q—at specified time intervals.

Based on the results presented in Figure 4. It was observed that both unmodified hydrogels and hydrogels incorporated with protein particles showed swelling properties. However, it may be noticed that the addition of albumin increased the swelling ability of tested materials. The highest swelling ratios were calculated for sample 1.0_protein. Introduction of higher amount of this modifier—i.e., 3.0 mg/1.0 g hydrogel (sample 3.0_protein)—also increased hydrogels’ swelling ratios compared to their values calculated for unmodified materials, although they achieved lower values than in the case of 1.0_protein sample. The increase in the swelling ratios of modified polymers compared to unmodified ones results from the fact that the proteins increase the affinity for water [39]. These biomolecules fulfil specific biological functions and actively interact with molecules of water. Polar groups on the surface of the proteins form hydrogen bonds with water while charged amino acid side chains interact with its molecules electrostatically. Therefore, the occurrence of these interactions probably affected the increase in the swelling capacity of proteins-containing hydrogels [40]. In the case of 3.0_protein sample an increase in the swelling ability compared to the ability of unmodified material was observed however not to the extent as in the case of 1.0_protein sample. This may result from too many protein particles in the hydrogel matrix which—despite their interactions with water—may also constitute a steric hindrance to the penetrating liquid. The possibility of the synthesis of hydrogels showing various swelling abilities depending on the amount of the modifying agent constitutes undoubtedly an advantage because this allows for the preparation of personalized materials for specific applications (e.g., the dressing material should absorb larger amount of liquid to accelerate the wound healing process whereas the material intended for cosmetics industry should give up as much water as possible ensuring at the same time the most effective skin hydration). Therefore, it is worth emphasizing that developed materials show a wide application potential.

#### 2.2.2. Results of Incubation of Hydrogels in Selected Liquids

**Figure 5:**
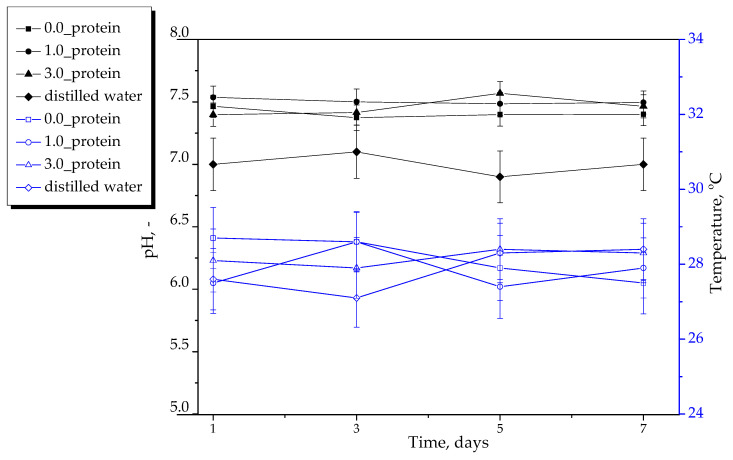
Results of hydrogels’ incubation in distilled water (*n*—number of repetitions, *n* = 3).

**Figure 6:**
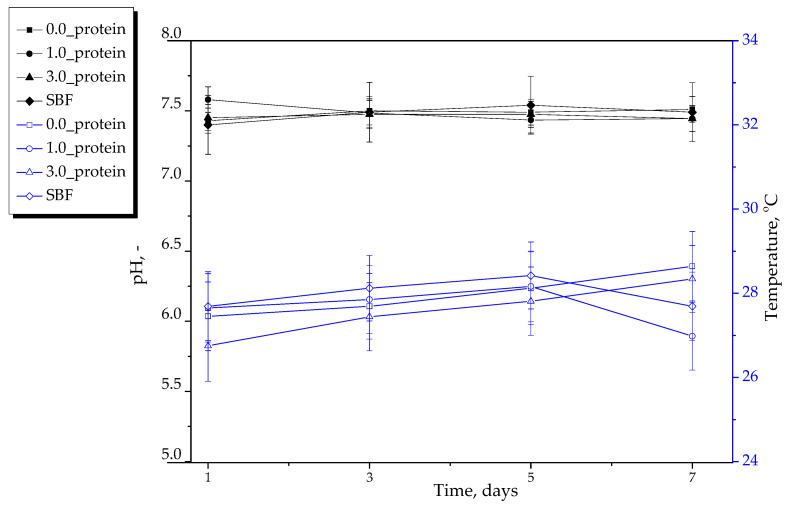
Results of hydrogels’ incubation in SBF (*n*—number of repetitions, *n* = 3).

**Figure 7:**
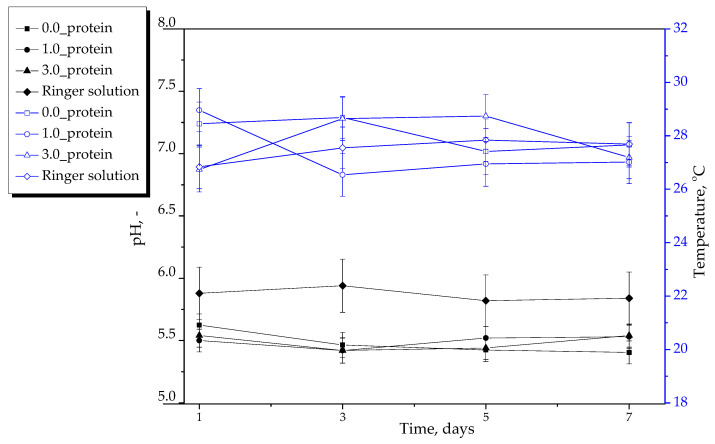
Results of hydrogels’ incubation in Ringer liquid (*n*—number of repetitions, *n* = 3).

In Figure 5, Figure 6 and Figure 7, results of incubation studies are presented. Each graph shows the changes in pH and the temperature of tested media in the presence of the tested hydrogel materials. The incubation was performed for 7 days wherein the mentioned parameters were measured every two days. The studies were conducted in triplicates and their results are shown as average values from performed measurements with corresponding standard deviations (SD, given as error bars).

An important step of the studies on the development of materials intended for biomedical purposes is to perform in vitro investigations reflecting the environment of human organism. One example of such experiments are incubation studies conducted in media corresponding to the in vivo conditions. For this purpose, the studies were performed in simulated body fluid (SBF) whose ionic composition and pH are similar to the human blood plasma and in Ringer liquid, i.e., a balanced polyionic liquid isotonic with human blood. Moreover, the study was also performed in distilled water treated as a reference liquid. As reference samples, the pH and temperature measurements of incubation media without hydrogel were also treated. In the case of all samples, any significant changes in measured parameters compared to their values determined for reference samples were not reported. The pH values were constant during the whole incubation period. In the case of the material with unreacted reagents between polymer chains within its network the noticeable change in pH of incubation medium might be observed but such a change was not reported. Probably, in the case of the material containing unreacted reagents in its structure, their elution could result in the change in pH of the incubation medium. However, such a change was not observed. Thereby it was concluded that the hydrogels were adequately crosslinked. The hydrogels showed also the stability in SBF and Ringer liquid and did not degrade in these liquids. Thus, the results obtained suggested that developed hydrogels did not interact with the components of the simulated physiological liquids, were inert towards them and stable during 7-day incubation.

#### 2.2.3. Analysis of the Chemical Structure of Hydrogels Using FT-IR Spectroscopy

**Figure 8:**
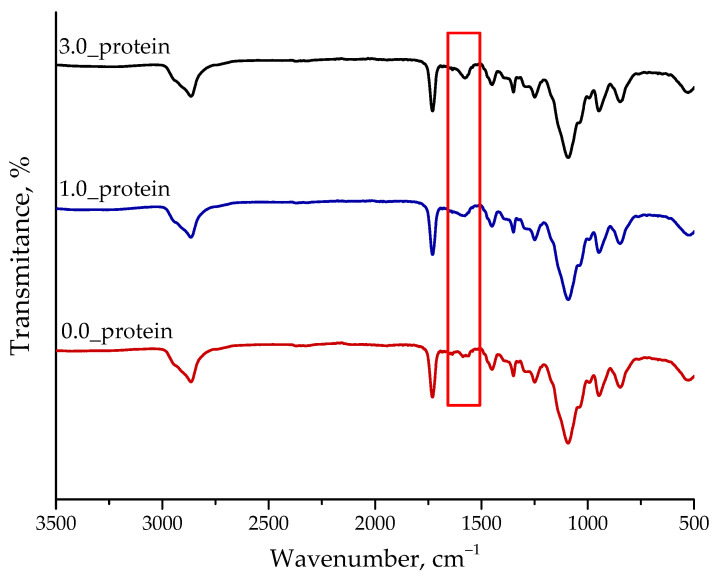
FT-IR spectra of unmodified hydrogel (sample 0.0_protein, red) and hydrogels containing protein particles (i.e., samples: 1.0_protein (blue) and 3.0_protein (black)).

In Figure 8 the FT-IR spectra of the hydrogels are presented.

Spectroscopic analysis allowed to determine the absorption bands characteristic for substances used during the synthesis of the hydrogels. In the case of unmodified materials, the absorption bands characteristic both for crosslinking agent and natural polymers, i.e., chitosan and gelatin, were noticed. The absorption band at 1660 cm^−1^ corresponding to the primary amine (-NH_2_), and the band at 1080 cm^−1^ characteristic for C-N stretching vibrations characteristic for chitosan were also noticed while they were previously reported also by Zhang et al. and Hajiabbas et al. Additionally, the presence of absorption bands at 1645 cm^−1^ corresponding to the amide I (C=O stretching vibration) characteristic for gelatin were also observed [41,42]. The particular attention should be paid to the absorption band marked with a red frame in Figure 8. Such a band was observed at approximately 1540 cm^−1^ and is characteristic for amide bonds II corresponding to the stretching vibrations of C-N bond coupled with the bending vibrations of N-H group. The mentioned interactions are characteristic for peptide bond occurring in amino acids consisting in albumin, i.e., a modifier of tested hydrogels [43]. This band may also be observed in the case of the FT-IR spectrum of 0.0_protein sample so this one does not contain albumin while its presence results from the application of gelatin as a component of the polymer matrix. Nonetheless, it should be emphasized that in the case of hydrogels modified with albumin particles an increase in the intensity of this band may be observed which may indicate the presence of the mentioned modifying agent—i.e., albumin particles in the tested materials.

#### 2.2.4. Results of SEM Imaging of Hydrogels

**Figure 9:**
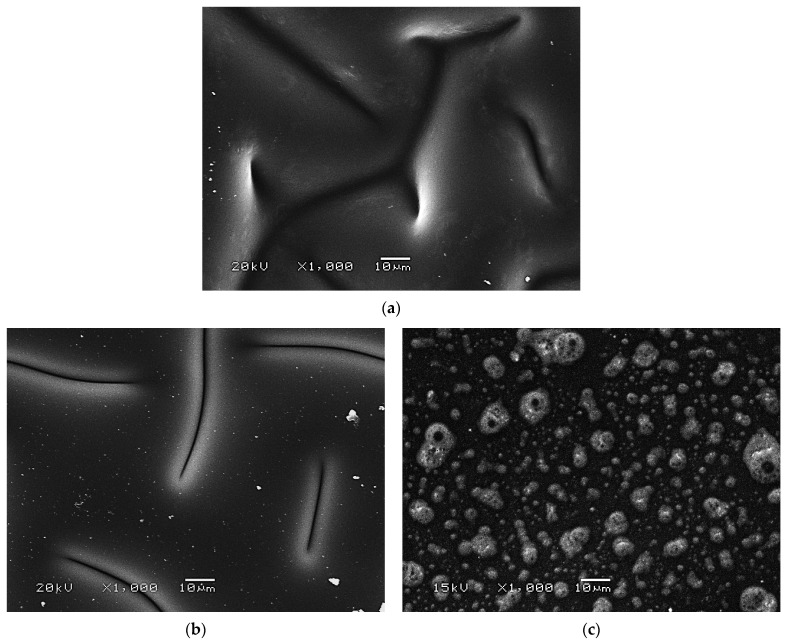
SEM images of unmodified hydrogel (**a**) and hydrogels containing protein particles: sample 1.0_protein (**b**) and sample 3.0_protein (**c**).

In Figure 9 hydrogel images obtained using scanning electron microscopy are shown.

In above-presented SEM images, the surface morphology of both unmodified hydrogel materials and materials incorporated with protein particles was shown. In the case of material modified with lower amount of the protein particles—i.e., 1.0_protein—its surface morphology seems to be very similar to the surface morphology of unmodified hydrogel. Referring to the results of DLS analysis, the average diameter of the particles obtained was approximately 35 nm therefore this protein is not visible in the SEM images whereas the surface morphology of modified materials seems to be homogeneous. However, the introduction of higher amount of this modifier, i.e., 3.0 mg/1 g hydrogel (3.0_protein), leads probably to the formation of the protein agglomerates which may be observed in Figure 9c. Based on the results obtained, it may be stated that the system showing greater homogeneity was obtained for sample containing 1.0 mg albumin/1.0 g hydrogel (so sample 1.0_protein) wherein the preparation of a homogeneous material with greater amount of the modifying substance (so sample 3.0_protein) requires probably an additional step including the disintegration of protein agglomerates (using e.g., ultrasounds or homogenizer).

#### 2.2.5. Studies on the Wettability of Hydrogels

As part of the research, the wetting angles measurements of distilled water were performed while the study was conducted with simultaneous recording of the behavior of the drop of the wetting liquid during its first contact with tested materials. Moreover, the total surface free energy for each hydrogel sample was also calculated. The results obtained during the analysis are presented in Table 1.

The surfaces whose wetting angle is lower than 90° are defined as hydrophilic. According to the previous reports, the cells show higher tendency to adhere to hydrophilic surfaces. Thus, the increase in the surface hydrophilicity may result in a better surface protein adsorption and, consequently, better cell adhesion and proliferation [44,45]. As demonstrated by Wei et al., fibroblast spreading and adhesion is also more effective for hydrophilic surfaces [46]. Thus, considering the potential application of developed materials for biomedical purposes, i.e., as hydrogel dressings, it is very important to provide conditions conducive to growth and development of skin and epidermal cells including fibroblasts. The wetting angles of all analyzed materials were lower than 90° which indicates hydrophilic surfaces, well-wettable with by water. Furthermore, it was observed that modification of hydrogels with protein particles resulted in the decrease of the wetting angles of such modified materials thus increasing their hydrophilic nature. This probably results from the occurrence of previously mentioned interactions between polar surface groups of proteins and water molecules resulting in formation of hydrogen bonds [40]. In terms of potential use of the hydrogels, the results of wettability measurements constitute undoubtedly their advantage. It is assumed that developed materials with hydrophilic surface will be an adequate substrate to cell adhesion and growth thus supporting regeneration processes.

#### 2.2.6. Mechanical Characteristics of Hydrogels including Determining Their Tensile Strength and Percentage Elongation

**Figure 10:**
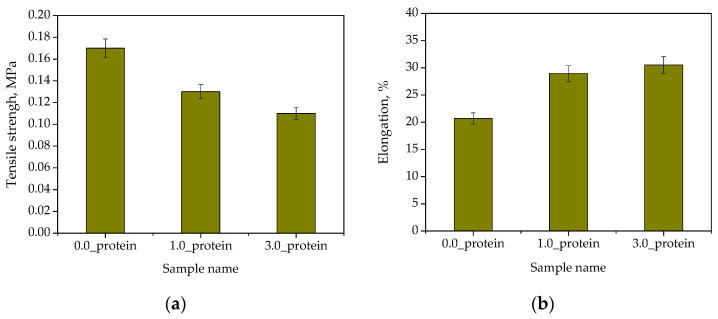
Results of the mechanical evaluation of unmodified hydrogels and hydrogels containing protein particles: tensile strength (**a**) and percentage elongation (**b**) (*n*—number of repetitions, *n* = 3).

Next, studies aimed at verifying the mechanical properties of developed materials while the main attention during the analysis was paid to defining the impact of the protein content on these properties. The study was performed in triplicate. The results are presented in Figure 10 in the form of bar charts showing the average values with corresponding standard deviations (SD, shown as error bars).

Considering the results of performed analysis, it may be concluded that the modification of hydrogels with albumin particles resulted in the decrease in their tensile strength and simultaneous increase in their percentage elongation. Introduction into the reaction mixture additional reagent with simultaneous use the same amounts of the photoinitiator and crosslinking agent may lead to the formation of the polymer matrix with a more relaxed polymer network. This, in turn, is due to a certain dilution of the reaction mixture. As a result, the materials showing lower tensile strength and higher elasticity (thus higher percentage elongation) are prepared. Moreover, the hydrogels were designed so as to be useful as dressing materials. Thus, the preparation of the material demonstrating adequate elasticity is very important wherein improving this property by the hydrogel modification is undoubtedly additional advantage of these materials.

#### 2.2.7. Studies on Determining the Release Profile of Albumin from Hydrogel Materials

**Figure 11:**
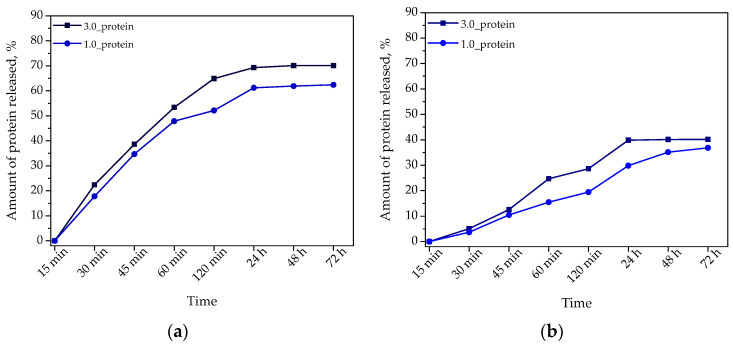
Results of the protein release from hydrogels in the environment with pH = 2.0 (**a**) and pH = 7.4 (**b**).

Below, in Figure 11, the results of the studies on the release of albumin particles from hydrogels placed in environments with different pH values are presented.

Albumin is a protein showing a row of properties favorable in terms of biomedical applications. This protein improves the cell adhesion and proliferation, exhibits anti-thrombotic and anti-inflammatory properties and, importantly, may be easily combined with various therapeutics thus constituting a promising drug carrier [47]. Therefore, an extremely important was to develop hydrogel materials demonstrating the ability to release albumin. Analyzing the results of studies aimed at determining the release profile of albumin from hydrogels as a function of time it may be observed that the release occurred both in acidic and slightly alkaline environment whereas the more effective process took place in an environment with pH = 2.0. In citric acid solution, the maximum amount of released protein particles was approximately 70% while in the case of the second tested environment—this was approximately 40%. When the hydrogel is placed in an acidic environment, in which hydrogen ions occur, the protonation of NH_2_ groups of chitosan takes place. Next, as a result of electrostatic interactions homonymous ions repel each other which, in turn, results in a loosening of the chains of polymer network. Consequently, molecules of the release medium penetrate easier the hydrogel matrix wherein albumin particles present inside the matrix are easier released from the hydrogel.

Albumin shows a tendency to accumulate near the neoplastic tissue [48]. Therefore, this protein may constitute a promising carrier of cytostatics. Thus, considering the carrier potential of albumin, the developed materials providing its effective release in acidic conditions is an innovative hydrogel material with high application potential. According to the literature reports, local acidification of the environment near the neoplastic tissue occurs [49]. Therefore, the use of the developed hydrogels as dressings supporting cancer treatment might be a favorable and interesting solution. Firstly, the hydrogel dressing showing swelling properties absorbs the wound exudate near the neoplastic tissue while at the same time in such acidic environment the release of albumin—which simultaneously might transport cytostatic drugs—is provided.

#### 2.2.8. In Vitro Biological Studies on Hydrogels via the MTT Reduction Assay

**Figure 12:**
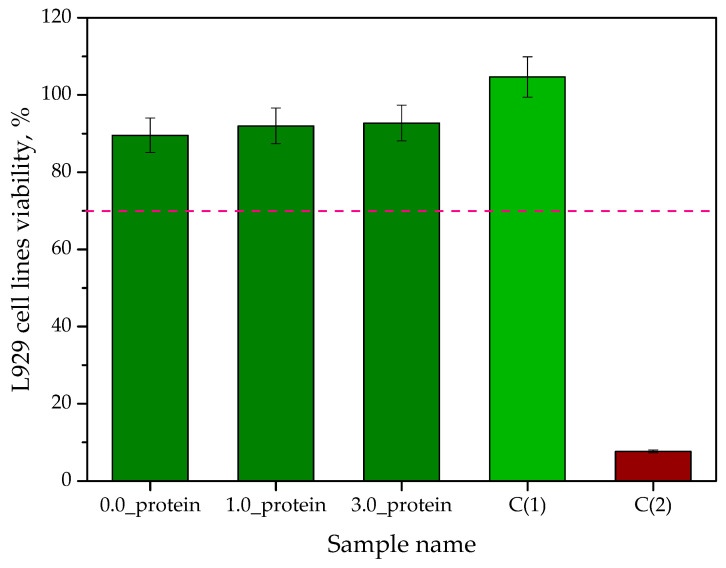
The viability of mouse fibroblasts (L929 cell lines) in the MTT reduction assay (*n*—number of repetitions *n* = 3).

Results of MTT reduction assay are presented in Figure 12. The study was performed in triplicate while the results are presented as average values with corresponding standard deviations (SD, showing as error bars). The symbol C(1) denotes cells in the culture medium, while C(2) denotes cells incubated with a 1% solution of phenol showing a strong cytotoxic effect.

The evaluation of the cytotoxic effect of materials intended for biomedical purposes performed in in vitro conditions is an extremely important introduction for further, more advanced, biological and toxicological studies. A series of detailed biological analyses allows for the verification of the safety in the use of a given material which translates into its classification or rejection for further research stages including in vivo analyses. MTT reduction assay is one of the basic tests in the biological evaluation of candidate materials for medical devices. According to EN ISO 10993-5: 2009 standard [50], a material is considered non-toxic towards an analyzed cell line when the survival rate of the cells treated with this material for 24 h is above 70% (it has been marked with red dotted line in Figure 12). In the case of the developed hydrogels, this requirement was met while the murine fibroblast viability was 89.545 for the unmodified hydrogels, and for modified 91.98% (1.0_protein) and 92.73% (3.0_protein), respectively. It may be observed that the addition of the albumin particles increased the viability of tested cell lines. The changes are slight but consistent with the results presented by other researchers. For example, Gonzalez Porto et al. proved that the addition of albumin increased the viability of Schwann cells (cells building the myelin sheaths around peripheral nerves) [51]. In turn, Ashman et al. indicated that the albumin stimulates the proliferation of proximal tubular epithelial cells [52].

#### 2.2.9. Analysis of the Pro-Inflammatory Activity of the Hydrogels

**Figure 13:**
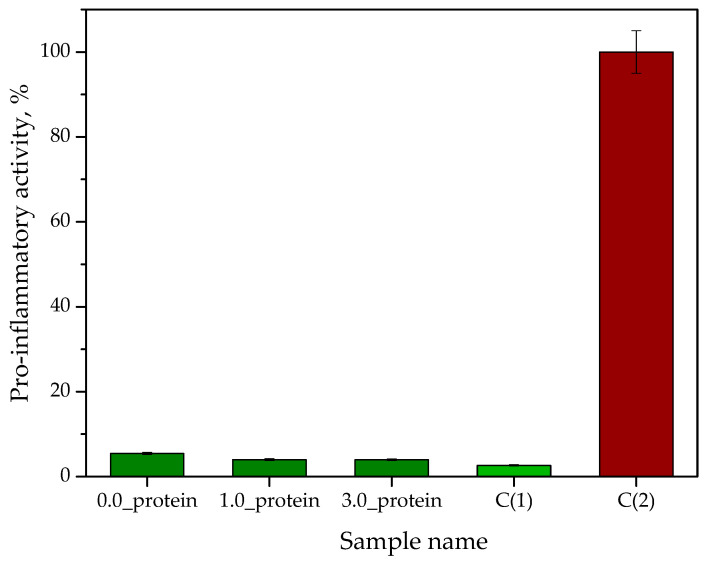
Results of analysis of hydrogels’ pro-inflammatory activity (*n*—number of repetitions *n* = 3).

Results of the analysis of the pro-inflammatory activity of hydrogels are presented in Figure 13. The study was performed in triplicates while the results are presented as average values with corresponding standard deviations (SD, showing as error bars. The symbol C(1) denotes cells in the culture medium, while C(2) denotes cells incubated with lipopolysaccharide (LPS) *E. coli* (i.e., a pro-inflammatory agent).

NF-κB (nuclear factor kappa-light-chain-enhancer of activated B cells) is one of the key transcription factors. It is present in almost all animal cells and is involved in the rapid response of cells to stimuli such as viral or bacterial infections, oxidative stress and a number of cytokines. NF-κB is a complex that plays a role in the regulation of immune response. It is also involved in physiological and pathological states including apoptosis, carcinogenesis and inflammatory processes [53]. Induction of the NF-κB leads to the secretion of SEAP (secreted embryonic alkaline phosphatase) into the culture medium, in which its presence may be detected by colorimetric analysis [54]. The described dependance made it possible to verify the pro-inflammatory activity of THP-1XBlueCells™ treated with the developed hydrogel materials. Thus, based on the performed study, it was demonstrated that the hydrogels showed no pro-inflammatory activity wherein the results obtained are similar to the control sample consisted of unstimulated cells (suspended in the culture medium). Considering the potential application of the hydrogels as dressings supporting the skin regeneration processes and acting simultaneously as carriers of the therapeutic agents the results obtained are very satisfactory and prove a well-chosen research direction.

## 3. Materials and Methods

### 3.1. Materials

Chitosan (deacetylation degree 75–85%, high molecular weight), gelatin (from porcine skin, gel strength 300, Type A), diacrylate poly(ethylene glycol (crosslinker, average molecular weight Mn = 700 g/mol, d = 1.120 g/mL), 2-hydroxy-2-methylpropiophenone (photoinitiator, d = 1.077 g/mL, 97%), phosphate buffered saline (PBS, tablets), potassium phosphate, and tris(hydroxymethyl)aminomethane (ACS reagent, 99.8%) were bought in Merck (Darmstadt, Germany). In turn, albumin (albumin egg powder), and hydrochloric acid (35–38%, d = 1.190 g/mL) were purchased in Avantor Performance Materials Poland S.A. (Gliwice, Poland).

### 3.2. Synthesis of Albumin Particles via the Salt-Induced Precipitation Process

In order to obtain protein particles, the salt-induced precipitation method was applied. As a salting out agent 2M K_3_PO_4_ was used wherein Tris-HCl buffer was selected to dissolve the protein formed. The albumin solutions in Tris-HCl buffer of various concentrations were prepared. The concentrations of the solutions of the reagents used for preparation of protein particles are given below in Table 2.

Obtained solutions were mixed in a volume ratio 1:1. The albumin solution was gradually dropped to the potassium phosphate solution with constant stirring. The protein solution was added dropwise at a rate of 1drop/s. The process was performed at room temperature at constant stirring (150 rpm), and after completion of the dropwise addition an intensive stirring (200 rpm) for 15 min was additionally applied. After mixing both reagents, the precipitated particles were centrifuged (15 min, 80 rpm), suspended in phosphate buffered saline (0.5 mL PBS buffer), and subjected to the physicochemical characteristics.

### 3.3. Synthesis of Chitosan/Gelatin-Based Hydrogels Incorporated with Albumin Particles by the Photopolymerization Process

The hydrogels were obtained via the UV-induced polymerization process. Firstly, 3% chitosan solution in 0.05% acetic acid solution and 2% aqueous solution of gelatin were prepared. Next, adequate amounts of the base solution (consisting of chitosan and gelatin solutions mixing in a volume ratio 1:1), suspension of albumin particles in PBS, crosslinker and photoinitiator were mixed, poured down to the Petri dishes (diameter 30 cm) and treated with UV radiation for 120 s. As a radiation source, the EMITA VP-60 lamp (power 180 W, λ = 320 nm) was applied. Detailed compositions of the hydrogels are presented below in Table 3.

After the synthesis the hydrogels were dried for 24 h at 37 °C and subjected to studies aimed at verifying their physicochemical properties.

### 3.4. Analysis of the Size of the Albumin Particles via DLS Technique

First analysis included determining the size of the albumin particles obtained via the salt-induced precipitation method. For this purpose, the particles were suspended in PBS and subjected to the DLS analysis. The study was performed using Zetasizer Nano ZS Malvern apparatus (Malvern Panalytical Ltd., Malvern, UK). Analysis was conducted at room temperature using the detector fixed at 173° while the results were obtained using CONTIN algorithm.

### 3.5. Characterization of Albumin Particles Using UV-Vis Spectroscopy

Optical properties (i.e., the light absorption ability) of protein particles were characterized via UV-Vis spectroscopy while the ThermoScientific Evolution 220 UV-Vis spectrometer (Thermo Fisher Scientific, Waltham, MA, USA) was used for this purpose. The study was carried out at room temperature.

### 3.6. Determining the Chemical Structure of Both Protein Particles and Hydrogels by FT-IR Spectroscopy

In order to determine the presence of characteristic functional groups in the structure of both albumin particles and hydrogels (unmodified and these incorporated with various amount of protein) FT-IR spectroscopy was employed. To perform the analysis, the Nicolet iS5 Thermo Scientific (Thermo Fisher Scientific, Waltham, MA, USA) spectrometer was used. Depending on the material, a drop of the suspension of albumin in PBS or dry hydrogel sample were subjected to the research. The FT-IR spectra were recorded within the range of 4000–550 cm^−1^ at a resolution of 4.0 cm^−1^. The measurements were conducted at room temperature.

### 3.7. Swelling Capability of Hydrogels

An important part of the experiments was characterizing hydrogels’ swelling ability. For this purpose, liquids simulating the environments occurring in human body, i.e., Ringer liquid (infusion liquid) and SBF liquid (simulated body fluid, isotonic to human blood plasma), and distilled water as a reference liquid were selected. Firstly, dry circular hydrogel samples (weighing approximately 1.0 g) were immersed in 50 mL of the mentioned liquids for 1 h. After this time, the samples were separated from the liquids, the excess liquid (unbound liquid) from the hydrogels surfaces was removed using a paper towel, and such swollen samples were weighed again. Such a procedure was repeated after 24 h and 72 h of swelling. The swelling ratios (*Q*) of hydrogels in selected liquids and after specific periods were calculated using the Equation (1) given below:

(1) $$Q=\frac{m-m_{0}}{m_{0}}$$

where: *Q*—swelling ratio, g/g; m—weight of swollen sample after specific period of time (i.e., after 1 h, 24 h or 72 h), g; $m_{0}$—weight of dry sample (before the study), g.

The study made possible to discuss the swelling ability of hydrogels depending on their composition (protein content), the absorbed medium, and the swelling time.

### 3.8. Incubation of Hydrogels in Simulated Body Liquids

Next study concerned determining the mutual interactions between the developed hydrogels and the simulated physiological liquids. For this purpose, hydrogel samples were immersed in selected liquids—i.e., SBF, Ringer liquid and distilled water—for 7 days. During this period the pH and the temperature of the incubation media were measured every two days via a multifunctional ELMETRON CX-701 (Elmetron, Zabrze, Poland) meter. The incubation was performed at 37 °C to simulate human body temperature.

### 3.9. Analysis of the Surface Morphology of Hydrogels via SEM Technique

The hydrogels’ surface morphology was evaluated using scanning electron microscope. Before the imaging, dry hydrogel samples were sputtered with gold. The analysis was performed at room temperature using a Jeol 5510LV (Jeol Ltd., Tokyo, Japan) microscope.

### 3.10. Studies on Hydrogels’ Mechanical Properties

Considering the potential application of developed materials, it is also important to verify their mechanical properties including such parameters as their tensile strength and elasticity. The knowledge of these parameters will allow to determine their application scope. The studies were conducted in line with ISO 527-2 type 5A and ISO 37 type 2 standards using the universal testing machine (Shimadzu, Kyoto, Japan). First step was to prepare paddle-shaped samples (by means of the ZCP020 manual blanking press) which were next placed between the jaws of the testing machine. During the analysis the jaws move apart from each other wherein the measurement was completed after the sample ruptured. The study was performed at room temperature. The tensile strength ($R_{m}$) was calculated using the Equation (2), while the percentage elongation (A) was determined based on the Equation (3):

(2) $$R_{m}=\frac{F_{m}}{S_{0}}$$

(3) $$A=\frac{\left(l_{u}-l_{0}\right)}{l_{0}}\times 100\%$$

where $F_{m}$—maximum strength, $S_{0}$—cross-sectional area of tested hydrogel in its initial state, $I_{u}$—measuring length after hydrogel rupture, $I_{0}$—measuring length of tested hydrogel in its initial state.

### 3.11. Wettability of Hydrogels

Next, analysis included determining the wettability of the hydrogels which was based on verifying the dynamic wetting angle between the hydrogel and tested liquid. The study was conducted by means of the Drop Shape Analyzer Kruss DSA100M measuring instrument (Gmbh, Hamburg, Germany) at room temperature. In order to perform the analysis, the hydrogel samples were placed into the platform inside the cell equipped with a fixed syringe with a wetting liquid (double distilled water). When the liquid dropped onto the hydrogel reached the equilibrium, the apparatus showed the calculated wetting angle. The measurements were performed with simultaneous recording the behavior of the drop during its contact with tested sample. Performed study allowed also to calculate the surface free energy. For this purpose, the Owens–Wendt method was applied.

### 3.12. Determining the Ability of Hydrogels to Release Albumin

An important aspect of the research was also to verify the ability of the release of albumin from developed hydrogels. The study was conducted in environments with various pH, i.e., in 2% citric acid solution (pH = 2.0) and in PBS buffer (pH = 7.4). For determining the presence of albumin in tested environments, UV-Vis spectroscopy was conducted. This was possible due to the presence of aromatic rings within the structure of amino acids included in albumin providing characteristic peak on the UV-Vis spectrum. Firstly, hydrogel samples weighing approximately 1.0 g were introduced into flasks containing 200 mL of tested environments. Then, the flasks were placed in the laboratory shaker (Hanchen ES-60E Temperature Controlled Incubator & Shaker Scientific Incu-Shaker Shaking Incubator, Tokyo, Japan) wherein the process was conducted at 37 °C. Next, after specific time periods 3 mL of tested solutions were given and subjected to the UV-Vis spectroscopy to verify the appearance of a peak characteristic for amino acids included in albumin what would testify about the release of this protein from hydrogels. Importantly, after each sampling the solutions were supplemented with 3 mL of PBS buffer or 2% citric acid solution to maintain the same volume of the tested environment. A similar procedure was also performed for 0.0_sample, i.e., sample unmodified with albumin particles. The results obtained for this sample constituted the reference results for the analysis of the modified hydrogels and enabled the exclusion of absorption bands that may also derive from chitosan or gelatin. Therefore, the results obtained referred only to the released albumin. The analysis was performed using the ThermoScientific Evolution 220 UV-Vis spectrometer (Thermo Fisher Scientific, Waltham, MA, USA).

### 3.13. In Vitro Biological Studies on Hydrogels via the MTT Reduction Assay

An important aspect of performed experiments was to evaluate the biological properties of developed hydrogels. As part of the study, in vitro biological analysis including determining the cytotoxicity of the materials via the MTT reduction assay was performed. The principle of the assay is to verify the viability of tested cells via defining their metabolic activity. For this purpose, the MTT reagent (i.e., 3-(4,5-dimethylthiazol-2-yl)-2,5-diphenyltetrazolium bromide; tetrazolium salt) is introduced into the medium with the cells (here: L929 murine fibroblasts). Metabolically active cells secrete into the culture medium various substances including, e.g., mitochondrial dehydrogenase wherein this enzyme converts the mentioned soluble MTT reagent into crystals of blue formazan. The crystals are next dissolved in an organic solvent (such as dimethyl sulfoxide (DMSO)) and such formed solution may be subsequently investigated via the UV-Vis spectroscopy. The concentrations of obtained solution corresponds then to the amount of the enzyme present in the tested medium and so gives the information on the amount of viable cells. The whole procedure of MTT reduction assay as well as the culture of L929 fibroblasts were described in detail in our previous publication [55].

### 3.14. Analysis of the Pro-Inflammatory Activity of the Hydrogels

The evaluation of the pro-inflammatory activity of developed hydrogels was performed using the human monocytic THP-1XBlueCells™ cell line (Invivogen, San Diego, CA, USA). The procedure applied was analogous to this one described in the previous publication [56]. Briefly, the cells were cultured in a culture medium RPMI-1640 with 10% heat-inactivated fetal bovine serum, L-glutamine (2 mM), and hydroxyethyl piperazineethanesulfonic acid (HEPES, 25 mM) supplemented with penicillin-streptomycin (100 U/mL–100 μg/mL) and additionally with selection factors, i.e., normocin (100 μg/mL) and blastocidin (10 μg/mL), incubated at 37 °C in 5% CO_2_. The THP-1XBlueCells™ cell line acts as an activation indicator for the NF-κB transcription factor. The presence of this factor may be confirmed indirectly by quantifying the amount of embryonic alkaline phosphatase (SEAP) excreted into the culture medium. In turn, the activation of THP-1XBlueCells™ cells induces a nuclear factor which then leads to the release of SEAP into the culture medium. The quantification of SEAP is possible using the Quanti-Blue™ reagent (Invivogen, San Diego, CA, USA). For the measurements, the prepared cell suspension with a density of 9 × 10^5^ cells/mL was placed in the wells of a 96-well plate (100 µL/well). Samples of the hydrogel materials were then introduced into the wells with THP-1XBlueCells™ cells and incubated under standard conditions for 24 h. Importantly, two controls were also performed, so C(1), i.e., a control sample containing cells in the culture medium and C(2), i.e., a control sample containing cells treated with lipopolysaccharide (LPS) *E. coli* O55:B5 (Sigma-Aldrich, Darmstadt, Germany) in a concentration of 1 μg/mL. After 24 h of incubation, the plates were centrifuged (10 min, 1400 rpm), and the supernatant obtained (20 µL) was transferred into the wells of another plate containing Quanti-Blue™ reagent (180 µL/well). Subsequently, after a 3 h of incubation, the tested suspensions were subjected to UV-Vis spectroscopy wherein the absorbance was determined at a wavelength of 620 nm using a Thermo Fisher Scientific multi-mode microplate reader.

## 4. Conclusions

- The applied synthesis methodology of protein carriers allowed to prepare albumin particles with nanometer sizes (less than 40 nm) showing low polydispersity.
- Both spectroscopic analyses performed—FT-IR and UV-Vis—confirmed the occurrence of the adsorption bands characteristic for the protein used during the carriers’ synthesis.
- Obtained protein carriers may be successfully applied as modifying agents of hydrogel materials based on such natural polymers as chitosan and gelatin.
- Hydrogels modified with protein particles showed higher swelling ability, higher elasticity and more hydrophilic surface compared to unmodified hydrogels.
- Both unmodified hydrogels and hydrogels incorporated with protein particles demonstrated stability during 7-day incubation in simulated physiological liquids.
- FT-IR spectroscopy confirmed the occurrence of additional adsorption bands characteristic for albumin on the FT-IR spectra of protein-modified hydrogels. However, SEM analysis indicated that in the case of sample containing 3.0 mg albumin/1.0 g hydrogel, the agglomeration of the particles may take place.
- Presented results indicated the possibilities of the potential application of developed materials as dressings supporting wound healing processes and delivering drugs via the protein carriers. It was demonstrated that the release of protein particles from hydrogels is more effective in an acidic environment—in these conditions approximately 70% protein was released wherein the release profile is prolonged.
- Considering the potential application of developed hydrogels as materials supporting the skin cancer treatment, their ability of effective releasing albumin in an acidic environment constitutes an advantage of these materials because this will enable or release this protein (combined with a chemotherapeutic drug) near the neoplastic tissue where the local acidification of the environment is observed.
- The results of MTT reduction assay confirmed the lack of cytotoxic activity of developed hydrogels towards L929 murine fibroblasts. The viability of tested cell lines was 89.54% for unmodified hydrogel while in the case of hydrogels containing albumin particles the values of this parameter was 91.98% (1.0_protein) and 92.73% (3.0_protein), respectively.
- Based on the analysis of pro-inflammatory activity, the negative immune response of THP-1XBlueCells™ cells incubated in the presence of the obtained hydrogels was excluded.
- Developed materials constitute an interesting solution with a potential for further research. Obtained results confirmed appropriately selected research direction.

## Figures and Tables

**Table 1:** Results of hydrogels’ wettability analysis.

Sample Name	Total Surface Free Energy, mJ/m^2^	Contact Angle, °	Image of Hydrogel during Its First Contact with Water
0.0_protein	50.72	68.75 ± 0.79	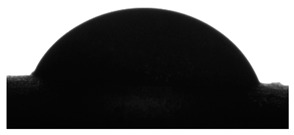
1.0_protein	49.16	64.51 ± 1.05	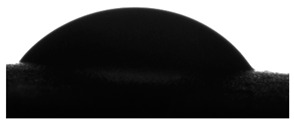
3.0_protein	48.96	62.28 ± 0.66	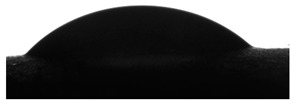

**Table 2:** Concentrations of the solutions used for preparation of protein particles.

Concentration of K_3_PO_4_, M	Concentration of Albumin, mg/mL
2.5	5
	10
	15
	20

**Table 3:** Compositions of hydrogel materials.

Base Solution, mL	Photoinitiator *, mL	Crosslinking Agent **, mL	Content of Albumin in Hydrogel Sample, mg Albumin/g Hydrogel	Sample Name
30	0.15	5.0	-	0.0_protein
			1.0	1.0_protein
			3.0	3.0_protein

* 2-hydroxy-2-methylpropiophenone ** diacrylate poly(ethylene glycol).

## Data Availability

Data sharing is not applicable for this article.
